# Dataset of aqueous humor cytokine profile in HIV patients with Cytomegalovirus (CMV) retinitis

**DOI:** 10.1016/j.dib.2016.06.050

**Published:** 2016-07-09

**Authors:** Jayant Venkatramani Iyer, Rupesh Agrawal, Tun Kuan Yeo, Dinesh V. Gunasekeran, Praveen Kumar Balne, Bernett Lee, Veonice Bijin Au, John Connolly, Stephen C.B. Teoh

**Affiliations:** a National Healthcare Group Eye Institute, Tan Tock Seng Hospital, Singapore; b Singapore National Eye Center, Singapore; c Institute of Molecular and Cell Biology (IMCB), Agency for Science, Technology and Research (A^⁎^STAR), Singapore; d Institute of Biomedical Studies, Baylor University, Waco, TX, USA

**Keywords:** Cytokines, CMV retinitis, Dataset, HIV, Luminex bead assay

## Abstract

The data shows the aqueous humor cytokine profiling results acquired in a small cohort of 17 HIV patients clinically diagnosed with Cytomegalovirus retinitis using the FlexMAP 3D (Luminex®) platform using the Milliplex Human Cytokine® kit. Aqueous humor samples were collected from these patients at different time points (pre-treatment and at 4-weekly intervals through the 12-week course of intravitreal ganciclovir treatment) and 41 cytokine levels were analyzed at each time point. CMV DNA viral load was assessed in 8 patients at different time points throughout the course of ganciclovir treatment. The data described herein is related to the research article entitled “Aqueous humor immune factors and cytomegalovirus (CMV) levels in CMV retinitis through treatment - The CRIGSS study” (Iyer et al., 2016) [1]. Cytokine levels against the different time points which indicate the response to the given treatment and against the CMV viral load were analyzed.

## **Specifications Table**

**Table:** 

Subject area	*Biology*
More specific subject area	*Cytomegalovirus retinitis in HIV patients*
Type of data	*Tables*
How data was acquired	*FlexMAP 3D (Luminex*®*) platform using the Milliplex Human Cytokine*® *kit*
Data format	*Raw*
Experimental factors	*Aqueous humor samples collected at different time points from HIV patients with Cytomegalovirus co-infection were analysed for the levels of 41 cytokines by Luminex bead based multiplex assay and Cytomegalovirus load by qPCR*
Experimental features	*Aqueous humor cytokine profiling was done by using FlexMAP 3D (Luminex*®*) platform using the Milliplex Human Cytokine*® *kit (EMD Millipore Corp.St. Charles, Missouri, U.S.A)*
Data source location	*Singapore*
Data accessibility	*Data is with this article*

## **Value of the data**

• The data presented in this article show a decreasing trend in the concentrations of G-CSF, MCP-1, IL-8, IL-10, IL-12p70, IFN-g, IP-10 and fractalkine in aqueous humor from HIV patients with CMV retinitis longitudinally through the course of the treatment.
• Analyzing the levels of these specific cytokines which involve in innate non-specific and Th1 driven anti-viral responses at different time points will allow clinicians to monitor the patient׳s response to the given treatment.
• The Luminex data identified the specific immune factors which may be used as disease prognostic markers for CMV retinitis in HIV patients.

## 1 Data

Luminex data on aqueous humor cytokine profiles from HIV patients with CMV retinitis revealed 9 cytokines G-CSF, IL-8, IFN-g, IP-10, fractalkine, IL-12p70, MCP-1, IL-10 and IL-6 showed a statistically significant negative correlation for samples analyzed longitudinally over the 4 times points (Table 1) and 9 cytokines MCP-1, IFN-g, IP-10, IL-8, fractalkine, RANTES, PDGF-AA, FLT-3L and G-CSF showed statistically significant positive correlation with CMV DNA viral load through course of treatment (Table 2).

## 2 Experimental design, materials and methods

Aqueous humor collected from 17 consecutive HIV-positive patients with clinically diagnosed with Cytomegalovirus retinitis over a one-year period. Prior informed consent was obtained from each patient. HIV diagnosis was confirmed by Western blot (Diagnostic Biotechnology HIV Blot 2.2), and CMV retinitis was diagnosed clinically, which is characterized by necrotizing retinitis with edematous or granular white borders, with or without associated hemorrhage, typically with minimal or no clinical vitritis or anterior chamber inflammation [2], [3]. Samples were collected at different time points (pre-treatment and at 4-weekly intervals through the 12-week course of intravitreal ganciclovir treatment) and 41 cytokine levels were analyzed at each time point with the FlexMAP 3D (Luminex®) platform using the Milliplex Human Cytokine® kit (EMD Millipore Corp.St. Charles, Missouri, U.S.A) following manufacturer׳s instructions [1]. CMV DNA viral load was assessed in 8 patients by qPCR at different time points throughout the course of ganciclovir treatment [1]. During the course of intravitreal ganciclovir treatment, aqueous humor cytokine levels were compared with each time point and also against the CMV viral loads of the tested samples [1].

## Figures and Tables

**Table 1:** Cytokine profile data of HIV patients with CMV retinitis at different time points.

sample_code	week	EGF	Eotaxin	FGF-2	Flt-3L	Fractalkine	G-CSF	GRO	IFN-a2	IFN-g	IL-10
001/001 TCL	0	0.06818586	0.14921911	0.74429298	1.58759873	2.44128657	2.34016639	2.22172713	1.20897852	1.26339933	1.42537117
001/002 TCL	4	0.06818586	0.15228834	0.74429298	1.45636603	2.25348339	2.00595229	1.04883009	0.59769519	0.20682588	0.83314711
001/003 TCL	8	0.06818586	0.14921911	1.09201847	1.15259408	1.98587536	1.68133171	1.19117146	−0.1487417	−0.1307683	0.64147411
002/001 HBM	0	0.06818586	0.14921911	0.74429298	0.82930377	0.71850169	1.5519377	1.38881141	−0.1487417	0.39794001	0.30963017
002/002 HBM	4	0.4785665	0.14921911	0.74429298	0.82930377	0.71850169	0.00860017	0.08990511	−0.1487417	0.09342169	0.04139269
002/003 HBM	8				1.14050804	1.81776363	0.96567197	0.77378644			
002/004 HBM	12	0.06818586	0.14921911	0.74429298	0.49554434	0.71850169	−0.236572	0.08990511	−0.1487417	−0.9208188	−2
003/001 LLH	2	0.06818586	0.14921911	0.74429298	0.49554434	0.71850169	1.19089172	0.08990511	−0.1487417	−0.9208188	−2
003/002 LLH	4	0.26951294	0.14921911	0.74429298	0.82930377	1.34262004	1.12385164	0.08990511	−0.1487417	−0.9208188	−0.9586073
003/003 LLH	8				0.90417437	1.50866436	0.90145832	0.08990511			
003/004 LLH	12	0.06818586	0.14921911	0.74429298	0.49554434	0.71850169	0.93550727	0.08990511	−0.1487417	−0.9208188	−2
004/001 CC	0	0.06818586	0.14921911	0.74429298	1.33825723	2.01598811	2.67107097	2.78841586	−0.1487417	1.58308537	0.90902085
004/002 CC	4	0.06818586	0.14921911	0.74429298	0.87506126	1.61626541	1.29688448	0.08990511	−0.1487417	−0.9208188	−2
004/003 CC	8	0.06818586	0.14921911	0.74429298	0.49554434	0.71850169	0.99255352	0.08990511	−0.1487417	−0.9208188	−2
004/004 CC	12	0.06818586	0.14921911	0.74429298	0.86332286	1.1383027	1.09795107	0.08990511	0.26481782	0.17318627	−0.0362122
005/001 KHW	0	0.06818586	0.14921911	0.74429298	0.94939001	1.71424591	1.52192224	0.55990663	−0.1487417	0.27184161	0.12385164
005/002 KHW	4	0.06818586	0.14921911	0.74429298	0.7084209	1.98587536	1.3881012	0.55990663	−0.1487417	−0.9208188	0.12385164
005/003 KHW	8	0.06818586	0.14921911	0.74429298	0.70586371	0.71850169	1.16435286	1.01577876	0.26481782	−0.5228787	−2
005/004 KHW	12	0.07918125	0.14921911	0.74429298	0.93701611	1.1383027	0.31386722	0.81557775	−0.1426675	−0.0861861	0.60530505
006/001 THP	0	0.06818586	0.14921911	0.74429298	0.49692965	0.71850169	1.36021461	0.08990511	−0.1487417	−0.0043648	0.44090908
006/002 THP	4	0.06818586	0.14921911	0.74429298	1.04883009	1.70663245	1.39828731	1.12287092	0.26481782	0.44870632	0.55870857
006/003 THP	10	0.06818586	1.00173371	0.74507479	1.07151381	1.53630587	1.43964843	1.01577876	0.462398	−0.0861861	0.64836001
007/001 TS	0	0.06818586	0.14921911	0.74429298	0.49554434	1.61626541	1.04178732	0.55990663	−0.1487417	0.20682588	−2
008/001 LMK	0	0.06818586	0.14921911	0.74429298	1.02407499	0.71850169	0.94743372	1.19645254	0.69108149	1.16672606	0.70070372
008/002 LMK	5	0.06818586	0.14921911	0.74429298	1.02407499	0.71850169	0.7084209	0.08990511	−0.1487417	0.26007139	0.57518784
008/003 LMK	9				1.10720997	1.70217195	0.18469143	0.99782308			
009/001 LFS	0	0.98811284	1.25285303	0.74429298	0.49554434	0.71850169	1.76342799	1.54678935	0.41830129	−0.9208188	0.98632378
010/001 SCL	0				1.50596352	2.19437557	1.53224464	1.60552052			
010/002 SCL	4				1.2828486	1.91803034	0.99387691	1.22582599			
010/003 SCL	8				1.02448567	1.76559406	0.372912	0.09342169			
010/004 SCL	12	0.61909333	0.14921911	0.74429298	1.61846649	0.71850169	0.86093662	0.08990511	1.07371835	0.20682588	0.88422877
011/001 TTS	0	0.8733206	0.14921911	1.70994802	1.56110138	0.71850169	1.62179922	1.62664827	−0.1487417	0.48572143	0.897077
012/001 AAK	0				1.26363607	2.28499439	1.55654371	0.99782308			
012/002 AAK	4				1.10720997	1.99268604	1.35907623	0.77378644			
012/003 AAK	8	0.66275783	0.14921911	1.83588073	1.22530928	0.71850169	1.2828486	1.66922387	1.22401481	−0.9208188	0.59769519
013/001 CKY	0	0.91539984	0.14921911	1.3881012	1.54802069	0.71850169	1.47683163	0.08990511	1.12645611	−0.9208188	0.94349452
014/001 LCC	0	0.8733206	0.14921911	0.74429298	0.49554434	0.71850169	1.58579901	1.43933269	1.28330123	0.97772361	0.9652017
015/001 SCS	0	0.50242712	0.14921911	0.74429298	1.80441206	2.33573865	1.62179922	1.74569923	0.51054501	1.06520613	1.42308196
015/002 SCS	4	0.68930886	0.14921911	0.74429298	1.57368369	2.42610443	0.42160393	0.08990511	0.51054501	0.50920252	0.78604121
015/003 SCS	8	0.61909333	0.14921911	1.14332713	0.49554434	2.28483664	0.97220284	1.35198946	−0.1487417	−0.9208188	0.59769519
016/001 CYC	0	0.06818586	0.14921911	0.74429298	0.49554434	0.71850169	0.78958071	0.08990511	−0.1487417	0.71180723	0.920645
017/001 LFS	0	0.07188201	0.14921911	0.74429298	1.70577821	2.51834282	1.98788961	0.08990511	0.77887447	0.01703334	0.9652017


sample_code	week	IL-12p40	IL-12p70	IL-13	IL-15	IL-17	IL-1a	IL-1b	IL-1ra	IL-2	IL-3
001/001 TCL	0	−0.1804561	−0.3665315	−0.091515	0.1903317	0.0374265	−0.0757207	−1.30103	0.3783979	−0.091515	−0.5228787
001/002 TCL	4	−0.1804561	−0.3665315	−0.091515	−0.0655015	0.0374265	−0.0757207	−1.30103	−0.552842	−0.2596373	−0.5086383
001/003 TCL	8	−0.1804561	−0.3665315	−1.39794	−0.4317983	0.0374265	−0.0757207	−1.30103	−0.552842	−1.69897	−0.5228787
002/001 HBM	0	−0.1804561	−0.3665315	−0.552842	−0.4317983	0.0374265	−0.0757207	−1.30103	−0.552842	−1.69897	−0.5228787
002/002 HBM	4	−0.1804561	−0.3665315	−0.79588	−0.4317983	0.0374265	−0.0757207	−1.30103	0.3783979	−1.69897	0.20951501
002/003 HBM	8										
002/004 HBM	12	−0.1804561	−0.3665315	−1.39794	−0.4317983	0.0374265	−0.0757207	−1.30103	−0.552842	−1.69897	−0.5228787
003/001 LLH	2	−0.1804561	−0.3665315	−1.39794	−0.4317983	0.0374265	−0.0757207	−1.30103	−0.552842	−1.69897	−0.5228787
003/002 LLH	4	−0.1804561	−0.3665315	−1.39794	−0.4317983	0.0374265	−0.0757207	−1.30103	0.3783979	−1.69897	−0.5228787
003/003 LLH	8										
003/004 LLH	12	−0.1804561	−0.3665315	−1.39794	−0.4317983	0.0374265	−0.0757207	−1.30103	−0.552842	−1.69897	−0.5228787
004/001 CC	0	−0.1804561	−0.3665315	0.23552845	0.22010809	0.0374265	−0.0757207	−1.30103	−0.552842	−1.69897	−0.5228787
004/002 CC	4	−0.1804561	−0.3665315	−1.39794	−0.4317983	0.0374265	−0.0757207	−1.30103	−0.552842	−1.69897	−0.5228787
004/003 CC	8	−0.1804561	−0.3665315	−1.39794	−0.4317983	0.0374265	−0.0757207	−1.30103	−0.552842	−1.69897	−0.5228787
004/004 CC	12	−0.1804561	−0.3565473	0.05307844	−0.4317983	0.0374265	−0.0757207	−0.2757241	0.69284692	−0.0555173	−0.5228787
005/001 KHW	0	−0.1804561	−0.3665315	−0.091515	−0.4317983	0.0374265	−0.0757207	−1.30103	−0.552842	−1.69897	−0.5228787
005/002 KHW	4	−0.1804561	−0.3665315	−1.39794	−0.4317983	0.0374265	−0.0757207	−1.30103	−0.552842	−1.69897	−0.5228787
005/003 KHW	8	−0.1804561	−0.3665315	0.02530587	−0.4317983	0.0374265	−0.0757207	−0.5228787	−0.552842	−0.4317983	−0.5228787
005/004 KHW	12	−0.1804561	−0.3665315	0	−0.4317983	0.0374265	−0.0757207	−1.30103	−0.552842	−0.0132283	−0.5228787
006/001 THP	0	−0.1804561	−0.3665315	−1.39794	−0.4317983	0.0374265	−0.0757207	−1.30103	−0.552842	−1.69897	−0.5228787
006/002 THP	4	−0.1804561	−0.3665315	0.13672057	−0.4317983	0.0374265	−0.0757207	−1.30103	−0.552842	0.06069784	−0.5228787
006/003 THP	10	−0.1739252	−0.3565473	0.13672057	−0.4317983	0.0374265	−0.0757207	−1.30103	−0.552842	−0.552842	−0.5228787
007/001 TS	0	−0.1804561	−0.3665315	−1.39794	−0.4317983	0.0374265	−0.0757207	−1.30103	−0.552842	−1.69897	−0.5228787
008/001 LMK	0	−0.1804561	−0.3665315	0.05307844	−0.4317983	0.0374265	−0.0757207	−1.30103	0.11394335	−1.69897	−0.5228787
008/002 LMK	5	−0.1804561	−0.3665315	−0.1191864	−0.4317983	0.0374265	−0.0757207	−0.3872161	0.11394335	−0.1023729	−0.5228787
008/003 LMK	9										
009/001 LFS	0	−0.1804561	0.59549622	−1.39794	0.63245729	0.04139269	0.52374647	−0.7212464	1.07518185	−1.69897	0.24551267
010/001 SCL	0										
010/002 SCL	4										
010/003 SCL	8										
010/004 SCL	12	−0.1804561	0.55266822	−1.39794	−0.4317983	0.0374265	−0.0757207	0.34439227	1.7294078	−1.69897	0.36735592
011/001 TTS	0	0.98944982	−0.3665315	−1.39794	0.63245729	0.26245109	0.54654266	0.37106786	1.17897695	−1.69897	0.78958071
012/001 AAK	0										
012/002 AAK	4										
012/003 AAK	8	−0.1804561	0.30103	−1.39794	0.49276039	0.0374265	−0.0757207	0.25767857	0.69810055	−1.69897	−0.5228787
013/001 CKY	0	1.15986785	−0.3665315	−1.39794	0.83821922	0.54157924	0.38560627	0.05307844	−0.552842	−1.69897	0.51054501
014/001 LCC	0	1.40568779	0.84633711	−1.39794	0.71850169	0.0374265	0.13672057	−1.30103	−0.537602	−1.69897	0.43296929
015/001 SCS	0	1.23628528	1.00732095	−1.39794	0.86687781	0.0374265	0.00860017	0.22271647	1.35372394	1.50270018	−0.251812
015/002 SCS	4	0.94448267	0.63447727	−1.39794	−0.2596373	0.0374265	0.31806333	−1.30103	−0.552842	−1.69897	−0.0409586
015/003 SCS	8	0.96754798	−0.3665315	−1.39794	0.58206336	0.0374265	0.81023252	−1.30103	0.63042788	−1.69897	0.66651798
016/001 CYC	0	−0.1804561	0.8122447	−1.39794	−0.4317983	0.0374265	−0.0757207	−1.2218487	0.63042788	−1.69897	0.65417654
017/001 LFS	0	−0.1804561	0.86746749	−1.39794	−0.4317983	0.0374265	−0.0705811	−1.30103	−0.552842	−1.69897	0.55266822


sample_code	week	IL-4	IL-5	IL-6	IL-7	IL-8	IL-9	IP-10	MCP-1	MCP-3	MDC
001/001 TCL	0	−0.154902	−0.2839967	2.26906908	0.09342169	1.61034071	−0.4814861	3.945474	4.09802004	1.23904909	0.80345712
001/002 TCL	4	−0.154902	−0.2839967	1.66171806	0.09342169	0.8142476	−0.537602	3.09118492	3.81717916	0.77742682	0.97726621
001/003 TCL	8	−0.154902	−0.552842	0.96614173	0.09342169	0.14921911	−1.30103	2.1465931	3.27102799	1.07809415	1.14488542
002/001 HBM	0	−0.154902	−0.3187588	1.14332713	0.09342169	1.11126251	−1.30103	2.72551144	3.81996947	0.64246452	0.97726621
002/002 HBM	4	−0.154902	−0.4436975	−0.30103	0.09342169	0.36548798	−1.30103	0.43296929	2.91307174	0.77742682	0.97726621
002/003 HBM	8			1.03261876		1.1906118		2.07430434			
002/004 HBM	12	−0.154902	−0.49485	0.42324587	0.09342169	−0.1191864	−1.30103	0.43136376	2.88695297	0.64246452	0.97726621
003/001 LLH	2	−0.154902	−0.49485	−0.3098039	0.09342169	−0.2596373	−1.30103	0.43136376	2.8650388	0.64246452	0.80345712
003/002 LLH	4	−0.154902	−0.49485	−0.3098039	0.09342169	−0.3098039	−1.30103	0.43136376	2.91981524	0.64246452	1.07554696
003/003 LLH	8			−0.3098039		0.13033377		1.61700034			
003/004 LLH	12	−0.154902	−0.552842	−0.3098039	0.09342169	−0.2596373	−1.30103	0.43136376	2.97407856	0.64246452	0.80345712
004/001 CC	0	−0.154902	−0.1611509	3.04329939	0.09342169	2.66043856	−1.30103	4.03528762	4.29654125	1.833657	0.97726621
004/002 CC	4	−0.154902	−0.4436975	0.09691001	0.09342169	0.15836249	−1.30103	1.86075712	3.06407219	0.64246452	0.80345712
004/003 CC	8	−0.154902	−0.552842	−0.3098039	0.09342169	0.0374265	−1.30103	0.72427587	2.95483082	0.64246452	0.80345712
004/004 CC	12	−0.154902	−0.091515	−0.3098039	0.09342169	−1.0457575	−1.30103	1.07261748	2.81554454	0.64345268	0.80345712
005/001 KHW	0	−0.154902	−0.6197888	1.34596154	0.09342169	1.15624619	−1.30103	2.88080216	3.68074513	0.99913054	1.07554696
005/002 KHW	4	−0.154902	−0.49485	1.41796964	0.09342169	1.24772783	−1.30103	2.02534683	3.36841706	0.64246452	0.97726621
005/003 KHW	8	0.04921802	−0.05061	−0.3098039	0.09342169	−0.2146702	−1.30103	1.11527759	2.78184151	0.64246452	0.89320675
005/004 KHW	12	−0.154902	−0.091515	−0.3098039	0.09342169	−0.0809219	−1.30103	1.97859133	2.74956627	0.64246452	0.80345712
006/001 THP	0	−0.154902	−0.4436975	0.5378191	0.09342169	0.59879051	−1.30103	2.16253454	3.19321089	0.94645227	0.97726621
006/002 THP	4	−0.154902	0.04532298	0.00432137	0.09342169	0.0211893	−1.30103	2.72288883	3.1522547	0.64345268	0.89320675
006/003 THP	10	−0.154902	0.04532298	−0.3098039	0.09342169	−0.2146702	−1.30103	2.05176975	2.96915955	1.13289977	1.32715451
007/001 TS	0	−0.154902	−0.49485	0.77232171	0.09342169	0.86569606	−1.30103	2.09373678	3.33157912	0.64246452	0.97726621
008/001 LMK	0	−0.154902	0.01283722	0.79726754	0.09342169	0.70926996	−1.30103	3.52255247	3.50514184	0.64345268	0.80345712
008/002 LMK	5	−0.0362122	0.01283722	0.20411998	0.09342169	−1.0457575	−1.30103	2.64097806	3.18045276	0.97034688	0.89320675
008/003 LMK	9			0.69019608		−1.0457575		2.59562847			
009/001 LFS	0	1.06069784	0.77742682	0.83821922	0.09342169	0.84695533	0.94743372	2.22891341	3.21189976	1.91174338	0.80345712
010/001 SCL	0			1.41514035		1.47011635		3.55502186			
010/002 SCL	4			1.01953168		0.90091307		2.28449841			
010/003 SCL	8			0.25767857		0.41329976		2.40450873			
010/004 SCL	12	0.82672252	0.5301997	0.8573325	0.66370093	0.1931246	0.81090428	1.98461731	3.36766336	1.89757211	1.40208935
011/001 TTS	0	−0.154902	−0.6382722	1.08849047	0.09342169	0.58433122	1.01619735	1.90493183	3.20959543	0.64246452	0.80345712
012/001 AAK	0			0.25767857		0.94299959		2.86605684			
012/002 AAK	4			−0.3098039		0.29446623		2.05876756			
012/003 AAK	8	−0.154902	−0.6382722	0.97818052	0.09342169	−0.8860566	0.52113808	1.36586222	3.05535922	2.02031984	1.75671216
013/001 CKY	0	−0.154902	−0.6382722	−0.3098039	0.09342169	−1.0457575	−1.30103	0.43136376	2.38261131	0.64246452	1.2664669
014/001 LCC	0	−0.154902	0.7411516	2.71449741	0.09342169	1.19838213	0.52113808	2.783203	4.00769829	1.97423528	1.37912415
015/001 SCS	0	−0.154902	−0.6382722	1.44388855	0.97451169	1.07445072	−1.30103	3.36878692	3.76524896	1.70294725	0.80345712
015/002 SCS	4	−0.154902	0.46834733	1.09131516	0.4578819	0.161368	−1	2.60677893	3.47019873	1.98802356	1.57379958
015/003 SCS	8	−0.154902	−0.6382722	1.51943419	0.09342169	−0.2676062	−0.4685211	2.87805623	3.55196328	0.64246452	0.80345712
016/001 CYC	0	−0.154902	0.56584782	0.9106244	0.09342169	−1.0457575	0.72672721	2.26583157	3.2646809	1.91839734	0.80345712
017/001 LFS	0	−0.154902	1.83480205	1.58478338	0.86569606	−0.05061	1.01072387	1.67715052	2.91091242	1.92484763	1.61204174


sample_code	week	MIP-1a	MIP-1b	PDGF-AA	PDGF-BB	RANTES	sCD40L	TGF-a	TNF-a	TNF-b	VEGF
001/001 TCL	0	1.13735411	1.38863397	1.4740705	1.34654856	1.5344069	−0.1307683	0.19589965	0.39445168	−0.853872	0.920645
001/002 TCL	4	1.04336228	1.07554696	1.65214961	1.18383904	1.34537373	0.76937733	0.19589965	0.36921586	−0.0457575	1.70969387
001/003 TCL	8	0.18469143	−0.49485	0.95904139	0.51188336	0.46538285	−0.1366771	0.19589965	−0.091515	−0.853872	1.74686763
002/001 HBM	0	0.18469143	1.01494035	0.20682588	0.51188336	0.39967372	0.39619935	0.19589965	−1	−0.853872	0.70156799
002/002 HBM	4	0.18469143	0.76342799	−1.2218487	0.75891189	0.33845649	−0.1307683	0.19589965	−1.154902	−0.853872	0.70156799
002/003 HBM	8		1.20655604	−1.2218487							
002/004 HBM	12	0.18469143	−0.49485	−0.4436975	0.51188336	0.30319606	−0.1366771	0.19589965	−1.154902	−0.853872	0.70156799
003/001 LLH	2	0.18469143	−0.49485	0.19865709	0.51188336	0.30319606	−0.1366771	0.19589965	−0.3665315	−0.853872	1.66679868
003/002 LLH	4	0.18469143	−0.49485	0.36172784	0.51188336	0.22530928	−0.1366771	0.19589965	−0.3098039	−0.853872	0.70156799
003/003 LLH	8		1.00689371	0.28103337							
003/004 LLH	12	0.18469143	−0.49485	0.59217676	0.51188336	0.38560627	−0.1366771	0.19589965	−1.09691	−0.853872	0.70156799
004/001 CC	0	1.11159852	1.3332457	1.31428866	1.34654856	1.09342169	−0.1366771	0.19589965	−0.0705811	−0.853872	0.70156799
004/002 CC	4	0.18469143	−0.49485	0.95520654	0.51188336	0.39967372	−0.1366771	0.19589965	−1.154902	−0.853872	0.70156799
004/003 CC	8	0.18469143	−0.49485	0.7176705	0.51188336	0.26717173	−0.1366771	0.19589965	−0.6777807	−0.853872	0.70156799
004/004 CC	12	0.56229286	0.41161971	0.48000694	0.51188336	0.48855072	−0.1366771	0.19589965	−0.1249387	−0.2441251	1.48015073
005/001 KHW	0	0.76342799	1.15715444	0.99694925	0.51188336	0.30319606	−0.1366771	0.19589965	−0.2924298	−0.853872	1.18355453
005/002 KHW	4	1.03302144	0.96754798	0.75966784	0.51188336	0.38560627	−0.1366771	0.19589965	−0.4202164	−0.79588	1.59725628
005/003 KHW	8	0.33041377	−0.236572	0.35024802	0.51188336	0.462398	−0.1366771	0.19589965	0.00432137	−0.2441251	0.70156799
005/004 KHW	12	0.40823997	−0.49485	0.2380461	0.51188336	0.5563025	0.66931688	0.19589965	−0.0555173	0.05307844	0.70156799
006/001 THP	0	0.18469143	−0.49485	0.13033377	0.51188336	0.33845649	−0.1366771	0.19589965	−0.3279021	−0.853872	1.73495976
006/002 THP	4	0.18469143	0.34439227	0.77524626	0.51188336	0.63346846	0.88366144	0.19589965	0.10380372	−0.2441251	1.40312052
006/003 THP	10	0.64836001	0.77085201	0.9790929	0.51188336	0.51188336	0.66931688	0.19589965	−0.0268721	−0.0705811	0.70243054
007/001 TS	0	0.61909333	−0.4814861	0.87215627	0.51188336	0.33845649	−0.1366771	0.19589965	−0.2924298	−0.853872	1.68178377
008/001 LMK	0	0.92634245	−0.49485	0.59549622	0.51188336	0.81491318	−0.1366771	0.19589965	0.05690485	−0.853872	0.70156799
008/002 LMK	5	0.22530928	0.46686762	0.372912	0.51188336	0.56702637	−0.1366771	0.19589965	−0.0705811	−0.0043648	1.44420099
008/003 LMK	9		−0.49485	0.01283722							
009/001 LFS	0	1.15472821	−0.49485	0.65224634	0.51188336	1.56867098	−0.1366771	0.19589965	0.30319606	−0.853872	0.70156799
010/001 SCL	0		1.09096308	0.5865873							
010/002 SCL	4		0.92012333	0.46389299							
010/003 SCL	8		1.18184359	−0.3665315							
010/004 SCL	12	1.11958577	1.25647721	0.46089784	0.51188336	1.24625231	−0.1366771	0.19589965	−0.2076083	−0.853872	1.68868672
011/001 TTS	0	0.5301997	−0.49485	−1.2218487	0.51188336	1.25863728	1.00475116	0.19589965	−0.0604807	−0.853872	2.23741806
012/001 AAK	0		0.64836001	0.80753503							
012/002 AAK	4		0.88366144	0.64048144							
012/003 AAK	8	1.13767054	1.02366392	0.3283796	1.5794406	0.22271647	1.84192231	0.19589965	−1.154902	−0.853872	0.70156799
013/001 CKY	0	0.96661099	−0.49485	−0.0604807	0.51188336	0.71011737	−0.1366771	0.19589965	−1.154902	−0.853872	0.70156799
014/001 LCC	0	1.26670197	1.09898964	0.36172784	1.83809314	0.5899496	1.87145617	0.19589965	−0.7695511	−0.853872	0.70156799
015/001 SCS	0	1.47683163	0.67117284	0.70757018	1.64962689	1.37199091	−0.1366771	0.19589965	−1.154902	−0.853872	2.12616389
015/002 SCS	4	1.20736504	−0.49485	0.04921802	1.7846886	1.45909079	−0.1366771	0.19589965	−1.154902	1.1142773	0.70156799
015/003 SCS	8	1.1931246	−0.49485	0.11058971	0.51188336	0.41161971	1.65021035	0.31597035	−1.154902	0.78247262	0.70156799
016/001 CYC	0	1.14612804	−0.49485	0.31386722	1.38506978	0.87506126	1.85199175	0.19589965	−0.3767507	−0.853872	0.70156799
017/001 LFS	0	1.04099769	0.74272513	0.03342376	1.70926996	0.51054501	1.44279323	0.19865709	−1.154902	1.23829707	2.16348936

**Table 2:** Correlation of cytomegalovirus load with cytokine levels in HIV patients with CMV retinitis.

sample	virus_load_copies_per_ul	EGF	Eotaxin	FGF-2	Flt-3L	Fractalkine	G-CSF	GRO	IFN-a2	IFN-g	IL-10	IL-12p40	IL-12p70
001/001 TCL	833.1				38.67	276.22	218.84	166.6	16.16	18.32	26.61		
001/002 TCL	20.89		1.4		28.58	179.24	101.36	11.17	3.94	1.59	6.79		
001/003 TCL	2.898			12.34	14.19	96.78	47.99	15.51		0.72	4.36		
001/004 TCL	796.8				30.07	181.78	114.09	65.52	6.01	20.58	30.16		
001/005 TCL	47.73				29.33	189.31	354.73	40.61	2.44	5.37	8.62		
001/006 TCL	697.2				33.29	217.97	707.67	133.73	9.2	10.52	15.33		
001/007 TCL	12.02	2.77			30.8	135.41	172.06	50.39	0.73	4.05	13.43		
001/008 TCL	3.467				9.54	35.52	41.2			0.97			
001/009 TCL	12.16				25.09	135.41	128.07	25.67		2.48	1.78		
001/010 TCL	4.75				5.58		5.99	5.18		2.05	0.71		
001/011 TCL	11.04				3.98		2.55			1.23			
001/012 TCL	20.67				4.8		7.29	5.18	0.35	0.73			
002/001 HBM	80.55				6.73		35.62	24.46		2.48	2.02		
002/002 HBM	2.575	2.99			6.73		1			1.22	1.08		
002/004 HBM	0.1												
002/005 HBM	0.1				5.09		1.85				0		
003/001 LLH	0.1						15.5						
003/002 LLH	0.1	1.84			6.73	21.99	13.28				0.09		
003/004 LLH	0.1						8.6						
003/005 LLH	0.1				3.28		2.14		0.35	0.06		0.67	
004/001 CC	1830				21.77	103.73	468.87	614.33		38.27	8.09		
004/002 CC	3.106				7.48	41.31	19.79						
004/003 CC	0.1						9.81						
004/004 CC	0.1				3.64	6.87	6.25		0.91	0.73	0.45		0.21
005/001 KHW	1513				8.88	51.77	33.24	3.61		1.85	1.31		
005/002 KHW	36.35				5.09	96.78	24.42	3.61			1.31		
005/003 KHW	0.1				2.53		7.29	5.18	0.91	0.14			
005/004 KHW	0.283	0.59			4.32	6.87	1.02	3.26	0.35	0.4	2.01		
005/005 KHW	0.1				2.92				0.35	0.23			
005/006 KHW	0.1			2.77	3.28		1.44		1.95	0.14	1.45	0.33	
006/001 THP	57.15				3.12		22.9			0.97	2.74		
006/002 THP	8.603				5.58	25.43	12.5	6.63	0.91	1.39	1.8		
006/003 THP	0.1		5.01	2.77	5.88	17.18	13.75	5.18	1.44	0.4	2.22	0.33	0.21
006/004 THP	0.1				2.92		4.42		2.45	0.9			
006/005 THP	0.1				2.53	14.09	10.48		1.95				
007/001 TS	812					41.31	10.99	3.61		1.59			
008/001 LMK	2481				5.28		4.42	7.85	2.45	7.33	2.5		
008/002 LMK	25.66				5.28		2.55			0.9	1.87		

sample	virus_load_copies_per_ul	IL-13	IL-15	IL-17	IL-1b	IL-1ra	IL-2	IL-3	IL-4	IL-5	IL-6	IL-8	IL-9	IP-10
001/001 TCL	833.1	0.79	1.53			2.37	0.79			0.5	185.79	40.75	0.31	8820.09
001/002 TCL	20.89	0.79	0.84				0.53	0.29		0.5	45.87	6.5	0.27	1233.61
001/003 TCL	2.898									0.26	9.23	1.39		140.13
001/004 TCL	796.8	0.79	3.22				0.53			0.46	140.26	19.08		8602.6
001/005 TCL	47.73	0.36								0.42	368.1	6.86	0.16	2117.78
001/006 TCL	697.2	0.47	0.84			1.19				0.38	541.89	30.52	0.08	6231.9
001/007 TCL	12.02	0.03	1.07				0.01			0.8	400.28	8.94	0.04	2574.54
001/008 TCL	3.467	0.14								0.46	7.84	2.84		381.88
001/009 TCL	12.16		0.36			2.37				0.46	58.8	7.09		1335.72
001/010 TCL	4.75	0.99			0.14		0.3		0.35	0.43	1.95	0.04		195.38
001/011 TCL	11.04	0.31								0.36	1.82			100.28
001/012 TCL	20.67	0.68					0.13			0.4	5.72			139.89
002/001 HBM	80.55	0.26								0.46	13.89	12.9		531.49
002/002 HBM	2.575	0.14				2.37		1.6		0.34	0.48	2.3		2.69
002/004 HBM	0.1									0.3	2.63	0.74		
002/005 HBM	0.1									0.26		1.3		6.69
003/001 LLH	0.1									0.3		0.53		
003/002 LLH	0.1					2.37				0.3		0.47		
003/004 LLH	0.1									0.26		0.53		
003/005 LLH	0.1	0.55					0.05			0.43				
004/001 CC	1830	1.7	1.64							0.67	1104.82	457.53		10846.43
004/002 CC	3.106									0.34	1.23	1.42		72.55
004/003 CC	0.1									0.26		1.07		5.28
004/004 CC	0.1	0.55			0.26	2.45	0.43			0.4				5.9
005/001 KHW	1513	0.79								0.22	22.16	14.31		759.96
005/002 KHW	36.35									0.3	26.16	17.67		105.99
005/003 KHW	0.1	0.52			0.14		0.17		0.55	0.43		0.29		6.51
005/004 KHW	0.283	0.49					0.48			0.4		0.41		47.59
005/005 KHW	0.1	0.55					0.09			0.4				17.44
005/006 KHW	0.1	0.52		0.85	0.03	0.64	0.22			0.4				30.05
006/001 THP	57.15									0.34	3.43	3.95		145.37
006/002 THP	8.603	0.68					0.56			0.54	0.49	0.51		264.14
006/003 THP	0.1	0.68					0.13			0.54		0.29		56.32
006/004 THP	0.1	0.19			0.08	0.64	0.13			0.34				19.39
006/005 THP	0.1	0.34				4.87	0.39		0.75	0.32				
007/001 TS	812									0.3	5.9	7.32		124.07
008/001 LMK	2481	0.55				0.64				0.51	3.13	2.55		1665.4
008/002 LMK	25.66	0.37			0.2	0.64	0.39		0.45	0.51	0.79			218.74

sample	virus_load_copies_per_ul	MCP-1	MCP-3	MDC	MIP-1a	MIP-1b	PDGF-AA	PDGF-BB	RANTES	TNF-a	TNF-b	VEGF	sCD40L
001/001 TCL	833.1	12531.97	17.32		13.7	24.45	29.77	22.19	34.21	2.46		8.31	0.72
001/002 TCL	20.89	6564.14	5.97	9.47	11.03	11.88	44.87	15.25	22.13	2.32	0.88	51.23	5.86
001/003 TCL	2.898	1866.48	11.95	13.94			9.08		2.9	0.79		55.81	
001/004 TCL	796.8	11320.09	23.09	9.47	15.55	18.26	16.74	22.19	18.88	2.75		51.23	9.19
001/005 TCL	47.73	7733.45	14.47	13.94	3.68	2.29	19.74	8.14	33.24	1.16		44.75	
001/006 TCL	697.2	11508.62	11.95	17.47	12.27	21.52	31.01	5.72	36.15	1.97		31.94	5.86
001/007 TCL	12.02	7448.1	14.47	25.82	10.09	8.15	16.18		32.91	0.76			2.47
001/008 TCL	3.467	3510.31		9.47	4.26		4.24		3.8	0.66	0.51	37.72	4.18
001/009 TCL	12.16	9984.12	8.82	6.35	1.52		15.71	3.24	4.46	0.74		12.05	
001/010 TCL	4.75	1273.15	3.66	6.57	0.83		2.31		1.62	0.36	0.27		
001/011 TCL	11.04	859.69					0.91		1.96	0.36			
001/012 TCL	20.67	980.45					2.22		1.53	0.4			
002/001 HBM	80.55	6606.45		9.47		10.33	1.59		2.49	0.08			2.47
002/002 HBM	2.575	818.58	5.97	9.47		5.78		5.72	2.16				0.72
002/004 HBM	0.1	770.8		9.47			0.34		1.99				
002/005 HBM	0.1	864.1		9.47		2.69	1.18		1.83	0.56		33.92	
003/001 LLH	0.1	732.87					1.56		1.99	0.41		46.41	
003/002 LLH	0.1	831.39		11.88			2.28		1.66	0.47			
003/004 LLH	0.1	942.04					3.89		2.41	0.06			
003/005 LLH	0.1	174.19	2.19				0.82		1.44	0.36	0.13		
004/001 CC	1830	19794.33	68.16	9.47	12.91	21.52	20.6	22.19	12.38	0.83			
004/002 CC	3.106	1158.95					9		2.49				
004/003 CC	0.1	901.2					5.2		1.83	0.19			
004/004 CC	0.1	326.97	2.19		1.81	1.28	1.5		1.53	0.36	0.27	15.09	
005/001 KHW	1513	4794.5	9.96	11.88	5.78	14.34	9.91		1.99	0.49		15.24	
005/002 KHW	36.35	2335.68		9.47	10.77	9.26	5.73		2.41	0.36	0.14	39.54	
005/003 KHW	0.1	302.55		3.9	1.06	0.28	1.11		1.44	0.49	0.27		
005/004 KHW	0.283	280.88			1.27		0.86		1.79	0.43	0.55		2.33
005/005 KHW	0.1	261.29					1.13		1.44	0.36	0.27		
005/006 KHW	0.1	213.4		3.9			1.29		1.62	0.43	0.55	22.51	
006/001 THP	57.15	1560.29	8.82	9.47			1.33		2.16	0.45		54.3	
006/002 THP	8.603	709.93	2.19	3.9		1.09	2.97		2.14	0.62	0.27	12.64	3.81
006/003 THP	0.1	465.71	6.78	10.61	2.21	2.94	4.76		1.62	0.46	0.41	2.51	2.33
006/004 THP	0.1	293.49	2.19			0.5	0.61		1.62	0.51			
006/005 THP	0.1	187.35	3.66	5.34			1.42		1.44	0.4	0.06		
007/001 TS	812	2145.73		9.47	4.14	0.31	7.43		2.16	0.49		48.04	
008/001 LMK	2481	1599.96	2.19		4.21		1.96		3.26	0.56			
008/002 LMK	25.66	757.56	4.66	3.9	0.83	1.46	1.17		1.83	0.41	0.48	13.89	
